# Enriched environment remedies cognitive dysfunctions and synaptic plasticity through NMDAR-Ca^2+^-Activin A circuit in chronic cerebral hypoperfusion rats

**DOI:** 10.18632/aging.203462

**Published:** 2021-08-30

**Authors:** Xin Zhang, Xiaohua Shi, Jiaoqi Wang, Zhongxin Xu, Jinting He

**Affiliations:** 1 Department of Neurology, China-Japan Union Hospital of Jilin University, Changchun, China

**Keywords:** enriched environment, chronic cerebral ischemia, vascular cognitive impairment, chronic cerebral hypoperfusion, NMDAR-Ca^2+^-ActA signal pathway

## Abstract

Chronic cerebral ischemia (CCI) is one of the critical factors in the occurrence and development of vascular cognitive impairment (VCI). Apoptosis of nerve cells and changes in synaptic activity after CCI are the key factors to induce VCI. Synaptic stimulation up-regulates intraneuronal Ca^2+^ level through N-methyl-D-aspartic acid receptor (NMDAR) via induction of the activity-regulated inhibitor of death (AID) expression to produce active-dependent neuroprotection. Moreover, the regulation of synaptic plasticity could improve cognition and learning ability. Activin A (ActA), an exocrine protein of AID, can promote NMDAR phosphorylation and participate in the regulation of synaptic plasticity. We previously found that exogenous ActA can improve the cognitive function of rats with chronic cerebral ischemia and enhance the oxygenated glucose deprivation of intracellular Ca^2+^ level. In addition to NMDAR, the Wnt pathway is critical in the positive regulation of LTP through activation or inhibition. It plays an essential role in synaptic transmission and activity-dependent synaptic plasticity. The enriched environment can increase ActA expression during CCI injury. We speculated that the NMDAR-Ca^2+^-ActA signal pathway has a loop-acting mode, and the environmental enrichment could improve chronic cerebral ischemia cognitive impairment via NMDAR-Ca^2+^-ActA, Wnt/β-catenin pathway is involved in this process. For the hypothesis verification, this study intends to establish chronic cerebral hypoperfusion (CCH) rat model, explore the improvement effect of enriched environment on VCI, detect the changes in plasticity of synaptic morphology and investigate the regulatory mechanism NMDAR-Ca^2+^-ActA-Wnt/β-catenin signaling loop, providing a therapeutic method for the treatment of CCH.

## INTRODUCTION

Chronic cerebral hypoperfusion (CCH) is a common pathological state of the nervous system and an essential in the occurrence and development of vascular cognitive impairment (VCI) [1]. In the frontal lobe, hippocampus and other regions are more sensitive to ischemia and hypoxia, the changes in neuronal apoptosis and synaptic activity caused by CCH are more detectable, CCH is the crucial cause of VCI [2]. The synaptic activity was reported to participate in nerve cell signal transmission, memory formation, chronic pain and addiction, and improve nerve cell survival, which is called acquired neuroprotection [3]. Therefore, targeting the regulation of synaptic activity and plasticity is a new direction to improve CCH-induced cognitive impairment.

Environmental stimulation can affect the morphology and function of the brain and play an essential role in brain development, Hebb first proposed the concept of the enriched environment (EE) in 1947 [4], which is mainly composed of three main parts: sensory stimulation, cognitive activities and physical exercise [5]. Recent studies have found that the EE stimulation can regulate synaptic activity and plasticity, increase long-term potentiation (LTP), and improve the pathologically and physiologically cerebral cognitive dysfunctions [6, 7]. EE can enhance hippocampus-dependent spatial memory ability, improve age-related cognitive impairment, and reduce cognitive impairment in transgenic Alzheimer's mice through increasing beneficial stimuli from sensory, motor, space, and society [8, 9]. Moreover, EE can also improve spatial learning and memory functions in CCH rats [10].

The activity-dependent neuroprotection mediated by N-methyl-D-aspartate receptor (NMDAR) as an important part of acquired neuroprotection is an internal protective mechanism of neurons against external damage and stimuli [11, 12]. It is activated by synaptic activity, calcium signaling, and gene transcription [13, 14]. Genes that are activated by this process are collectively called activity-regulated inhibitors of death (AID) [15]. The Ca^2+^ signal induced by synaptic activity and transmitted to the nucleus is the primary pathway for synapses to communicate with the nucleus. Synaptic stimulation upregulates Ca^2+^ levels in neurons through NMDAR and initiates downstream AID gene expression, improving individual learning and memory capabilities through synaptic plasticity mechanisms [16, 17]. ActA increases the postsynaptic influx of Ca^2+^ in hippocampal neurons and participates in the regulation of synaptic plasticity through promoting the phosphorylation of NMDAR [18, 19]. The previous study found that ActA can effectively improve the cognitive dysfunctions of CCH rats [18, 19]. NMDAR can activate the Wnt signaling protein at hippocampal synapses and activate β-catenin through the classical Wnt signaling cascade to modify synaptic plasticity and LTP further, and β-catenin also plays an essential role in the formation and stability of synapses [20, 21].

This study intended to establish the CCH rat model, which was given with EE stimuli, to observe the changes of NMDAR-Ca^2+^-ActA signaling pathway and explore further whether EE can improve the CCH-induced cognitive dysfunctions the dual mechanisms of activity-dependent neuroprotection and synaptic plasticity.

## RESULTS

### EE improves VCI in CCH rats

**Figure 1:**
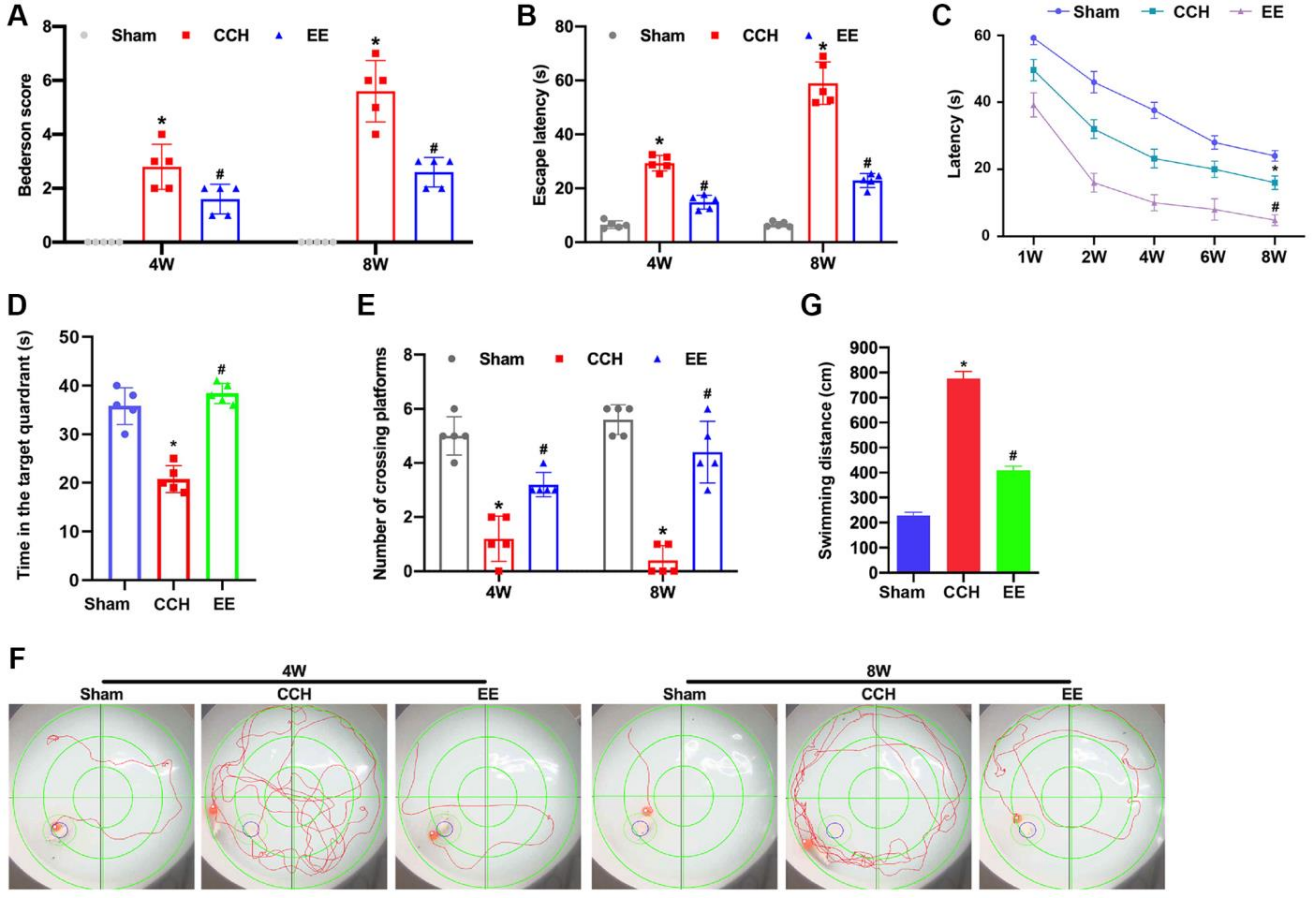
**EE improves VCI in CCH rats.** (**A**) Neurological impairment was measured by the Bederson scoring method. Memory performance was measured in the Morris water maze test. Neurological deficit scores. (**B**–**G**) Morris water maze test. The rats were put in the water randomly at any of the four quadrants. The incubation period in hidden platform is recorded as entering the water to climbing onto the platform. The training period is limited to one minute. If the rats could not find the hidden platform within one minute, it was recorded as 1 minute. After that, the rats were placed on the platform to rest for 1 minute to help them reposition the platform. The motion trajectories of rats were photographed and recorded by the system. The distance, duration, rest time, water intake times and water intake rate were statistically analyzed. The experiment was designed to assess the animals’ short-term memory and learning abilities. (**B**) The escape latency was shown. (**C**) The learning curve was shown. (**D**) The time in the target quadrants was shown. (**E**) The numbers of crossing platform were shown. (**F**) The swimming trajectories were shown. (**G**) The swimming distance was shown. ^*^p < 0.05, vs. Sham group; ^#^*p* < 0.05, vs. CCH group. Sham group, treated with an equal volume of vehicle; CCH group, chronic cerebral hypoperfusion and no treatment; EE group, CCH and treated with EE. *N* = 5. ^*^p < 0.05, ^#^*p* < 0.05.

The Bederson scoring system was used to determine the neurological damage in rats. After bilateral carotid artery ligation, the neurological function scores of rats gradually increased at 4 weeks and 8 weeks, while the neurological function of rats treated with EE was significantly improved (Figure 1A). Furthermore, the learning and memory ability of rats in each group was tested by Morris water maze test. The escape latency and the crossing times of the original platform were recorded as evaluation indicators. After the rat's bilateral arteries were ligated with for 4W, the escape latency and the time in the target quadrant were extended (Figure 1B–1D) and the crossing times of the original platform were decreased (Figure 1E). After 8 weeks of ischemia, the changes in escape latency and the crossing times of the original platform of CCH rats were more obvious, And the escape latency and the crossing times of the original platform of CCH rats were changed most significantly compared to the same indicators of CCH rats at 4w. While EE could significantly improve the performance of the rats' performance of spatial memory ability (Figure 1F and 1G), and the escape latency of CCH rats were gradually shortened, the crossing time of the original platform was increased after 4w and 8w of EE stimulation, and the impairment of learning and memory functions was repaired and nerve function damage was reduced in CCH rats.

### EE reduced the apoptosis rate of neurons in CCH rats

**Figure 2:**
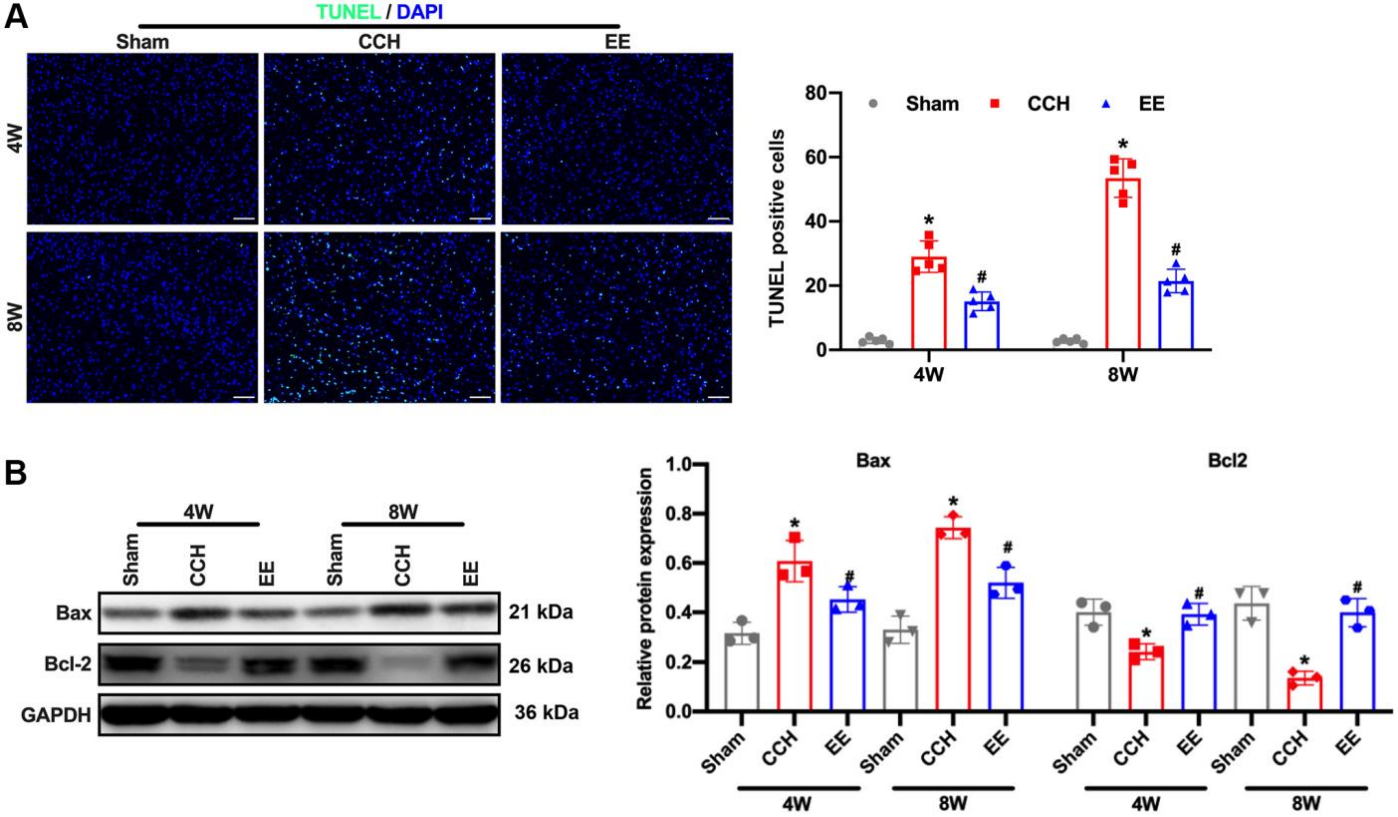
**EE alleviates brain damage in CCH rats.** (**A**) TUNEL staining was used to detect neuronal apoptosis in the frontal cortex and hippocampal in each group. Apoptosis-related proteins were detected by Western blot. TUNEL staining. Scale bar = 50 μm. ^*^p < 0.05, vs. Sham group; ^#^*p* < 0.05, vs. CCH group. *N* = 5. (**B**) Western blot. ^*^p < 0.05, vs. Sham group; ^#^*p* < 0.05, vs. CCH group. *N* = 3. Sham group, treated with an equal volume of vehicle; CCH group, chronic cerebral hypoperfusion and no treatment; EE group, CCH and treated with EE. ^*^p < 0.05, ^#^*p* < 0.05.

The results showed that the rats were ligated through bilateral common carotid artery for four or eight weeks, the hippocampal and frontal cortex of the rats were damaged, especially in the CCH group, Neuronal apoptosis was further observed by TUNEL staining. The results showed that the apoptosis rate of rats in the CCH group increased gradually with the extension of time, and the apoptosis rate was the highest at eight weeks (Figure 2A). However, under the enriched environment, the apoptosis rate of rats was significantly reduced, and the expression of Bax was reduced, and the expression of anti-apoptotic Bcl-2 was promoted (Figure 2B). These results demonstrated that the enriched environment could reduce the apoptosis rate of neurons in CCH rats and perform a protective role in brain injury.

### EE induces ActA to promotes Ca^2+^ influx in CCH rats

**Figure 3:**
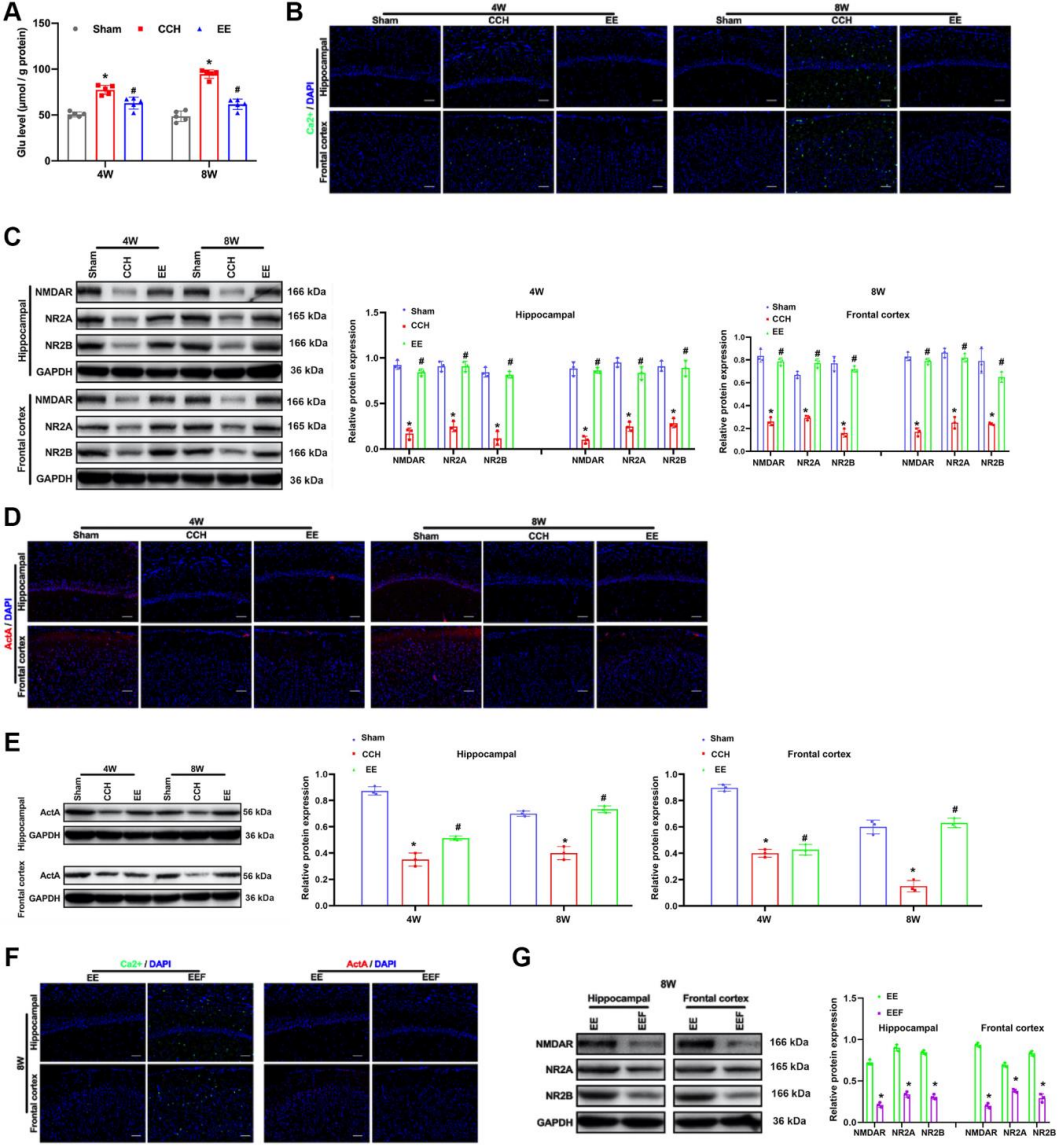
**EE induces ActA to promotes Ca^2+^ influx in CCH rats.** (**A**) Immunofluorescence staining and Western blot were used to detect the expression of cognitive-related proteins. Levels of L-glutamate (GLU). (**B**, **C**) The content of Ca^2+^ in the cerebral cortex and hippocampus. Levels of NMDAR, NR2A, NR2B. (**D**) The content of ActA in the cerebral cortex and hippocampus. (**E**–**G**) Levels of Act A. The content of Ca^2+^ and ActA block with FSH in the cerebral cortex and hippocampus. Levels of NMDAR, NR2A, NR2B block with FSH in the cerebral cortex and hippocampus. ^*^p < 0.05, vs. Sham group; ^#^*p* < 0.05, vs. CCH group. Sham group, treated with an equal volume of vehicle; CCH group, chronic cerebral hypoperfusion and no treatment; EE group, CCH and treated with EE. EEF group, CCH and treated with EE and FSH. *N* = 5. ^*^p < 0.05, ^#^*p* < 0.05.

When the rats were ligated through bilateral common carotid artery for four and eight weeks, the content of Glu in the rat brain tissues increased (Figure 3A). It was found that the fluorescence intensity of Ca^2+^ was lower in the CCH group, while the content of calcium ions in the cerebral cortex and hippocampus of the rats in a rich environment increased (Figure 3B). It is well known that NMDAR maintains the normal physiological function of neurons by regulating the influx of calcium ions. We detected the expression of NMDAR and its subunits NR2A and NR2B, and the results showed that enriched environment could up-regulate the expression of NMDAR, NR2A and NR2B in hippocampus and cortical areas (Figure 3C). At the same time, ActA expression was activated in hippocampus and cortex (Figure 3D, 3E). In order to verify that ActA upregulation of NMDAR in rich environment can improve intracellular Ca^2+^, ActA blocker (FSH) was administered to the rats in the enriched environment, and results showed that the effect of enriched environment was canceled (Figure 3F, 3G).

### EE activated ActA to improve synaptic morphology in CCH rats

**Figure 4:**
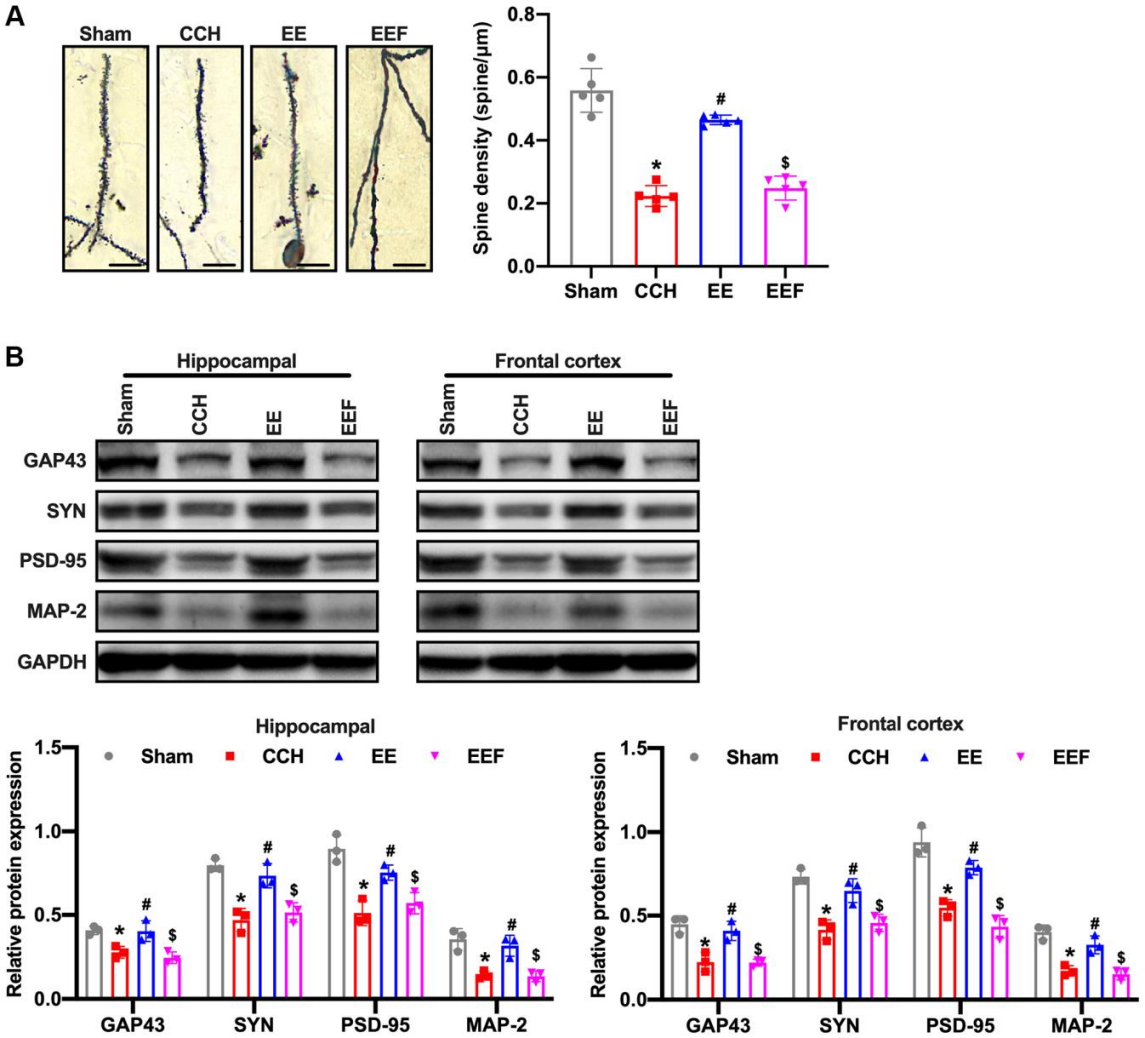
**EE improves synaptic morphology in CCH rats.** (**A**) Neuronal spine density changes were detected by Golgi staining in the hippocampal in each group. Western blot were used to detect the expression of synaptic plasticity associated proteins. Number and length of dendritic spines. *N* = 5. (**B**) Levels of GAP43, SYN, PSD-95, MAP-2. ^*^p < 0.05, vs. Sham group; ^#^*p* < 0.05, vs. CCH group. ^$^*p* < 0.05, vs. EE group. *N* = 3. Sham group, treated with an equal volume of vehicle; CCH group, chronic cerebral hypoperfusion and no treatment; EE group, CCH and treated with EE. EEF group, CCH and treated with EE and FSH. ^*^p < 0.05, ^#^*p* < 0.05, ^$^*p* < 0.05.

ActA has been reported to be involved in the regulation of synaptic plasticity by upregulating NMDARs, furthermore improving the cognitive function of rats with chronic cerebral ischemia. Firstly, the Golgi staining in hippocampus of brain tissues demonstrated that the head volume of the neuronal dendritic spines in CCH rats was gradually reduced, and the neck was elongated. After 8W breeding, the number of dendritic spines significantly decreased (Figure 4A). Western blot results showed that synaptic plasticity associated proteins GAP-43, Synapsin (SYN), Postsynaptic density 95 (PSD-95) and Microtubule Associated protein 2 (MAP-2) in CCH rats were decreased. However, the expressions of GAP43, SYN, PSD-95 and MAP-2 in the cortex and hippocampus of rats under enriched environment were increased (Figure 4B). However, the number of dendritic spines of neurons was not significantly decreased. The treatment of ActA antagonist attenuated the enriched environment-improved synaptic morphology in CCH rats, suggesting that enriching the environment could improve synaptic morphology in CCH rats by activating ActA.

### EE up-regulated ActA activates Wnt/β-catenin pathway involved in synaptic plasticity changes

**Figure 5:**
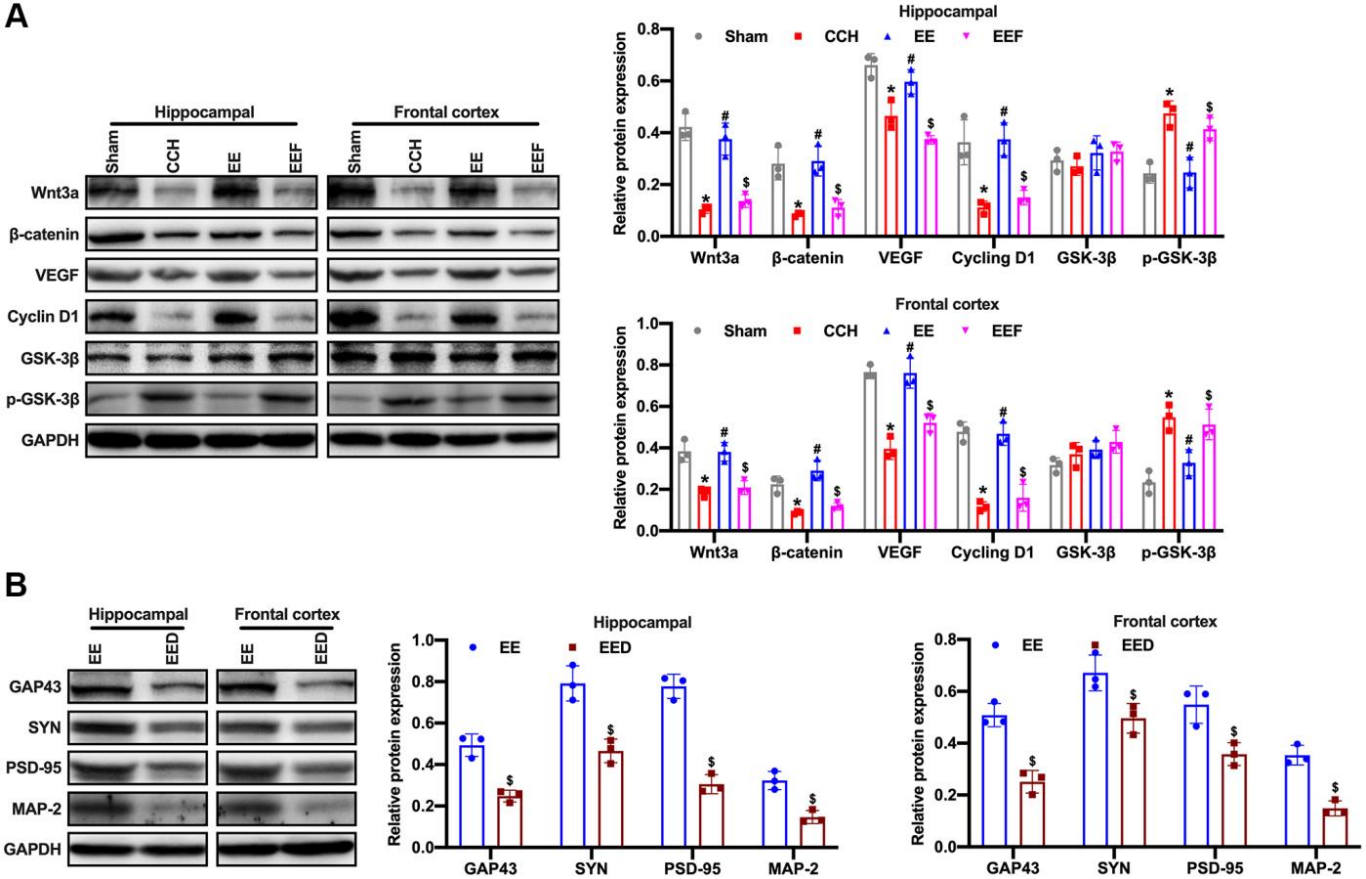
**EE activates Wnt/β-catenin pathway in CCH rats.** (**A**) Western blot were used to detect the expression of Wnt/β-catenin pathway associated proteins. Levels of Wnt3a, β-catenin, VEGF, CyclinD1, GSK-3β and p-GSK-3β. (**B**) Levels of GAP43, SYN, PSD-95, MAP-2. ^*^p < 0.05, vs. Sham group; ^#^*p* < 0.05, vs. CCH group. ^$^*p* < 0.05, vs. EE group. *N* = 3. Sham group, treated with an equal volume of vehicle; CCH group, chronic cerebral hypoperfusion and no treatment; EE group, CCH and treated with EE. EEF group, CCH and treated with EE and FSH. EED group, CCH and treated with EE and DKK-1. ^*^p < 0.05, ^#^*p* < 0.05, ^$^*p* < 0.05.

Studies have shown that activation of the Wnt/β-catenin signaling pathway can enhance neurogenesis and improve neurological function after focal ischemic injury. To verify whether the enriched environment could activate Wnt/β-catenin pathway by up-regulating ActA, the proteins related to the Wnt/β-catenin pathway were detected by Western blot. The results showed that the expression of positive regulatory proteins associated with the Wnt/β-catenin pathway in CCH rats was decreased, and the expression of Wnt3a, β-catenin as well as VEGF and CyclinD1 in the enriched environment was activated while up-regulating ActA. As administrated with ActA antagonist, the activation of the Wnt/β-catenin pathway was inhibited and the level of GSK-3 phosphorylation was increased (Figure 5A). To further investigate whether the Wnt/β-catenin pathway is involved in the change of synaptic plasticity, the Wnt/β-catenin pathway inhibitor DicKKopf-1 (DKK-1) was administered to rats with enriched environment, and the expressions of GAP43, SYN, SDD-95 and MAP-2 were decreased in the cortex and hippocampus of rats (Figure 5B). It was suggested that enriched environment up-regulated ActA, activated Wnt/β-catenin signaling pathway and regulated the synaptic plasticity changes.

### NMDAR antagonist (MK801) inhibits ActA expression and blocks the cognitive function of CCH rats in enriched environment

**Figure 6:**
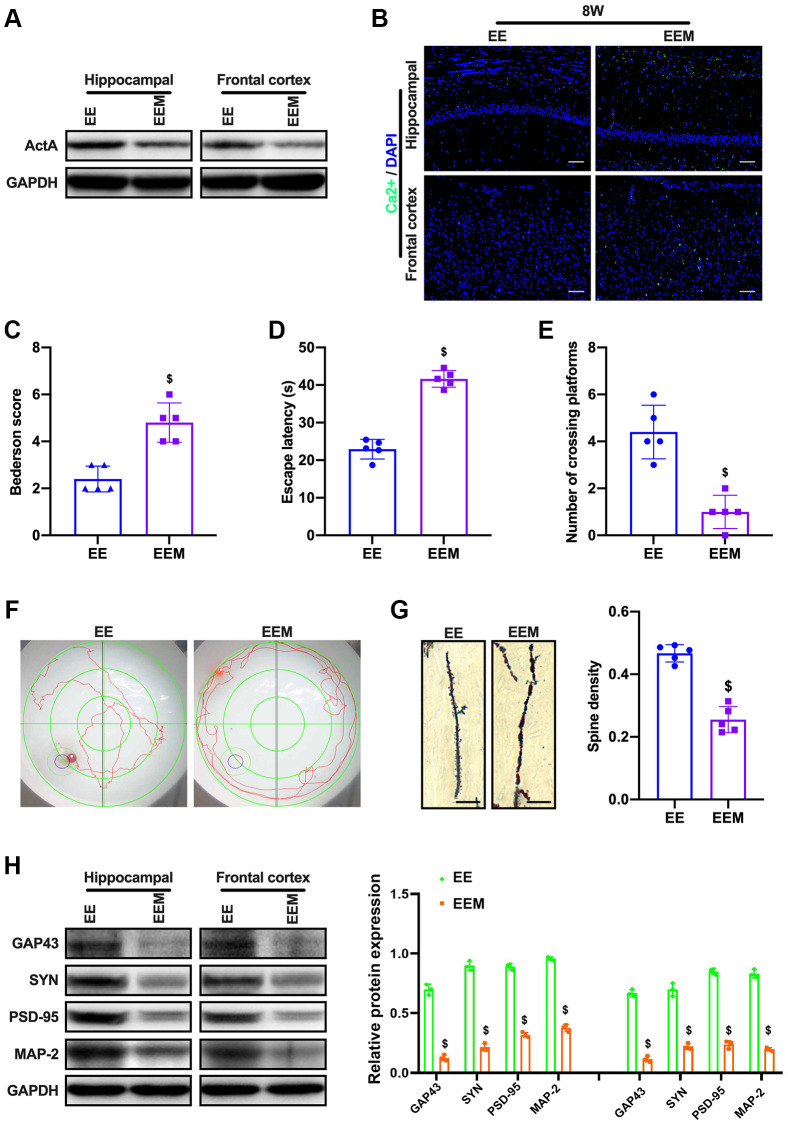
**MK801 blocks the cognitive function of CCH rats in EE.** (**A**) Immunofluorescence staining and Western blot were used to detect the expression of cognitive-related proteins and synaptic plasticity associated proteins. Neurological impairment was measured by the Bederson scoring method. Memory performance was measured in the Morris water maze test. Neuronal dendritic spines changes were detected by Golgi staining. Levels of ActA. (**B**, **C**) The content of Ca^2+^ in the cerebral cortex and hippocampus. Neurological deficit scores. (**D**–**F**) Morris water maze test. (**G**, **H**) Number and length of dendritic spines. Levels of GAP43, SYN, PSD-95, MAP-2. ^$^*p* < 0.05, vs. EE group. EE group, CCH and treated with EE. EEM group, CCH and treated with EE and MK801. *N* = 5. ^*^p < 0.05, ^#^*p* < 0.05, ^$^*p* < 0.05.

Then, we speculated that NMDAR-Ca^2+^ might have loop mode of action after CCI injury. To verify this hypothesis, CCH in the enriched environment was administered with NMDAR blocker MK801, then we demonstrated that ActA expression was inhibited (Figure 6A). The fluorescence intensity of calcium ion in brain tissue was consistent with that of CCH rats in the normal environment (Figure 6B). The cognitive function of the rats was not improved, the escape incubation period of the rats was prolonged and the crossing time of the original platform was decreased, and the learning and memory functions of the rats were still impaired (Figure 6C–6E). The number of neuronal dendritic spines decreased (Figure 6F). The expressions of GAP43, SYN, PSD-95 and MAP-2 in the cortex and hippocampus of rats were decreased (Figure 6G, 6H). Therefore, these results showed that MK801 inhibits the expression of ActA and blocks the protective effect of enriched environment on the cognitive function of CCH rats.

## DISCUSSION

Adequate cerebral perfusion plays an essential role in maintaining the normal function of the brain, while long-term and insufficient cerebral perfusion can lead to damage in the structure and function of nerve tissues [22]. Especially in the frontal lobe, hippocampus and other regions sensitive to ischemia and hypoxia, the nerve cell damage and apoptosis are more obvious. Patients often show impaired cognitive function, which is an important link in the occurrence and development of VCI. The main symptom of Vad is cognitive impairment, which is a leading cause of dementia in addition to Alzheimer's disease in the elderly [23]. Currently, the therapeutic method of Vad is still very limited, and thereby it is urgent to investigate the treatment of Vad.

Environmental stimulation can affect the morphology and function of the brain and play an important role in brain development [5]. Mice in the EE cage performed more motor, sensory, and cognitive stimuli than those in the normal cage [24]. Non-invasive environmental stimulation participated in reducing cognitive deficits and maintaining the integrity of brain tissue in rats’ brain injury, the cause of which is related to EE's alleviation of brain tissue injury and maintenance of neuronal structure integrity [25]. It is also accompanied by a series of plasticity responses in the brain, including cell proliferation, neuronal side outlays, new synaptic connections and changes in the density of synaptic vesicles, and changes in the plasticity-related proteins. Engineer et al. [26] confirmed that the number of primary auditory cortex (A1) neurons increased for two weeks after receiving EE stimulation in sexually mature rats. As EE stimulation and SE stimulation were given to the two groups of 21-week old cerebral ischemia rats respectively for 2 months, there were significant differences in the number of neurons in hippocampal CA1 region between the two groups, suggesting that EE has a protective effect on hippocampal pyramidal cells [27–29]. EE can increase the synaptic density in the cortex of rats with acute focal cerebral ischemia and induce the changes in the synaptic structure of the cortex and hippocampus. For transient focal cerebral ischemia rats, EE can also increase the neurogenesis of the dentate gyrus in the hippocampus and eventually reverse the cognitive impairment [28]. EE can reverse the cognitive impairment caused by acute or transient ischemic encephalopathy, however, the effect of EE on the cognitive impairment caused by CCH and its mechanism is still unclear. Therefore, this study is to explore the effects of EE on cognitive impairment and synaptic plasticity caused by CCH.

In this study, the nerve function of rats with bilateral carotid artery ligation was evaluated according to Bederson score, and the results demonstrated that the nerve function of the rats under the enriched environment was significantly improved. Under Morris water maze detection, after 4W and 8W of enriched environmental stimulation, the searching duration (escape incubation period) and searching walking path (swimming distance) of rats were gradually shortened, and the impaired learning and memory function was also repaired. TUNEL staining assay showed that the enriched environment reduced the apoptosis rate of neurons in the frontal cortex and hippocampus of rats. Western blotting results confirmed the conclusion. EE could significantly inhibit the expression of pro-apoptotic protein Bax and promote the expression of Bcl-2. These results suggest that the enriched environment could reduce the apoptosis rate of neurons in CCH rats and have a protective effect on brain injury.

AtcA is one of multifunctional cytokines belonging to the transforming growth factor-β (TGF-β) superfamily, was initially found to regulate the mammalian reproductive process and has the function in stimulating the release of follicle-stimulating hormone in the anterior pituitary, thus it was called as Activin [30]. ActA has been proved to regulate various cell functions, such as apoptosis, proliferation, differentiation, migration, etc [31]. Recent studies have shown that activators also play a role in neurons. Activin receptors are highly expressed in hippocampal structures, and activin has been shown to act as neurotrophic factor of basic fibroblast growth factor (BFGF), protecting neurons from damage [32–34]. The activity of NMDARs is equally important for functional development of neural circuits. Neonatal blocking of NMDAR alters γ oscillations, reduces the parvalbumin (PV) and the 67 kDa subtypes of glutamic decarboxylase (GAD67) and induces a variety of behavioral, cellular, and molecular changes that can be traced to GABA-destroying intermediate neurons [35]. NMDARs are the main molecules that control synaptic plasticity, memory function and LTP maintenance [36]. LTP is a form of synaptic plasticity that depends on activity, which is consider to play a key role in learning and memory formation [37]. As the neurotransmitter GLU is released to the synaptic junction, NMDARs infiltrate Ca^2+^ and increased the concentration of Ca^2+^ in postsynaptic neurons. Ca^2+^ influx triggers a series of intracellular signals, leading to the regulation of synaptic plasticity [38]. The Ca^2+^ influx induced by NMDARs depends not only on the frequency of synaptic transmission, but the relative Ca^2+^ permeability of NMDARs. The permeability of Ca^2+^ is mainly regulated by phosphorylation of Type 2 glutamate receptor N-methyl-D-aspartic acid isoform of Src family tyrosine kinases [39]. NMDARs are tetramer protein complexes that usually contain two NR1 subunits and two NR2 subunits [40]. ActA can act as an inducer of NMDAR activation in cultured hippocampal neurons, induce phosphorylation of NR2A and NR2B in time-dependent manner and enhance Ca^2+^ influx through NMDARs in response to L-glutamate stimulation [41]. In this study, NMDAR and its subunits NR2A and NR2B were detected, and the results showed that the enriched environment could up-regulate the expressions of NMDAR, NR2A and NR2B in hippocampus and cortex. At the same time, ActA expression was activated in the hippocampus and cortex. Western blot was used to detect GAP-43, SYN, PSD-95, and MAP-2. The results showed that the expression of synaptic plasticity associated proteins with CCH rats was decreased. However, the expressions of GAP43, SYN, PSD-95 and MAP-2 in the cortex and hippocampus of rats in EE group were increased, and the number of dendritic spines of neurons did not decrease significantly. As the ActA antagonist was administrated, the improvement of synaptic plasticity in CCH rats by enriching the environment was inhibited, suggesting that enriching the environment could improve the synaptic plasticity in CCH rats by activating Act A. However, we did not provided the hippocampus with zoom-in into the CAl area in Golgi staining and we will improve this issue in future investigations.

The Wnt/β-catenin signaling pathway has become a key pathway in neuronal self-renewal, proliferation, differentiation, maturation, and functional integration [42]. The Wnt/β-catenin signaling pathway has been shown to contribute to long-term enhancement of LTP, and dysfunction of the Wnt/β-catenin pathway is associated with the pathology of AD [43]. The upregulation of WNT/β-catenin is also related to the enhancement of the cognitive function of adult rats [44]. The study has proved that compared with sham group and 2VO Common Occlusion (2-VO), the sham + EE group and the 2VO + EE group showed a significant increase of Wnt3A expression after four weeks [10]. The expression of p-GSK-3 and β-catenin was significantly increased in sham + EE and 2VO + EE groups compared with SE rats (including sham operation and 2VO group). Enriched environment could activate Wnt/β-catenin signal pathway to improve learning and memory in rats with vascular dementia. In this study, it was found that the enriched environment could up-regulate ActA and activate the expression of Wnt/β-catenin pathway; Moreover, the activation of Wnt/β-catenin pathway was inhibited as ActA antagonist was administered. When Wnt/β-catenin pathway inhibitor DKK-1 was administered to rats in enriched environment, the expressions of GAP43, SYN, PSD-95 and MAP-2 in the cortex and hippocampus of rats were decreased. It was suggested that enriched environment could up-regulate ActA and activating Wnt/ β-catenin pathway involved in synaptic plasticity changes. Moreover, MK801 was administrated to CCH under enriched environment, however, ActA expression was inhibited. Therefore, we speculated that NMDAR-Ca^2+^-ActA- Wnt/β-catenin signal pathway might have a loop-acting mode after CCI injury.

EE can improve the function of many diseases in the central nervous system, including depression, stroke, traumatic brain injury and schizophrenia [5]. In Vad rats, EE can activate ActA through the NMDAR-Ca^2+^-ActA signaling pathways, and then activate Wnt/β-catenin signaling pathways, regulating synaptic plasticity, play an important role in learning behavior.

## MATERIALS AND METHODS

### Experimental animals and groups

The specific pathogen-free (SPF) Sprague Dawley (SD) rats, male, 260~300 g, were purchased from Beijing Vitalriver Laboratory Animal Technology Co., LTD. (License No. SCXK (Beijing) 201200070). Animal experiments were carried out in the laboratory animal facility of Jilin University. Rats were randomly divided into sham group (SHAM), chronic cerebral ischemia model group (CCH), enriched environment +CCH group, enriched environment + CCH+ activator inhibitor group, rich environment + CCH + Wnt/β-catenin pathway inhibitor group and enriched environment + CCH + N-methyl-D-aspartic acid receptor antagonist (mk801) group, with 5 rats per group. The rats were anesthetized by an intraperitoneal injection of 2% sodium pentobarbital (3 mg/kg) and placed in the supine position. The skin on the neck was prepared, and a midline incision was made to separate the bilateral common carotid arteries and vagus nerve. In the groups that were subjected to CCH, the bilateral common carotid arteries were permanently ligated at the distal end of the telecentric end with No. 4 surgical thread. The incision was then closed by layer-by-layer sutures. Gentamicin was injected subcutaneously to prevent infection, and the rats were returned to their home cages for feeding, followed by the indicated treatment in the different group. This study was approved by the Experimental Animal Welfare and Ethics Committee of China-Japan Union Hospital of Jilin University (IACUC No.2019116).

### Morris water maze experiment

Rats in each group were forced to swim in Morris water maze experiment, and rats’ cognitive, memory, learning and other neural functions were evaluated through measuring the spatial memory, spatial discrimination and working memory abilities of the animals. The rats were put in the water randomly at any of the four quadrants. The incubation period in hidden platform is recorded as entering the water to climbing onto the platform. The training period is limited to one minute. If the rats could not find the hidden platform within one minute, it was recorded as 1 minute. After that, the rats were placed on the platform to rest for 1 minute to help them reposition the platform. The motion trajectories of rats were photographed and recorded by the system. The distance, duration, rest time, water intake times and water intake rate were statistically analyzed. The experiment was designed to assess the animals' short-term memory and learning abilities. Memory retention tests were as followed: the platform was removed on day 6 and the rats were dropped into the water from the upper right quadrant. The system automatically records and analyzes the swimming time, cross-platform times and swimming distance within one minute of each quadrant. The residence time, swimming distance and crossing time (singular) of the original platform was recorded in the quadrant where the original platform was located.

### TUNEL assay

The paraffin-embedded sections of the brain tissues (*N* = 5) were treated with 50 μL TUNEL reaction solution and incubated at 37°C for 60 min. The sections were added with 50 μL streptomycin avidin-horseradish working solution, incubated in a box for 30 min, and the cell nuclei were stained with DAPI. The cells were dehydrated and the anti-fluorescence quenching blocks were sealed and photographed under a fluorescence microscope (Olympus DX51 fluorescence microscope, Olympus, Tokyo, Japan) at ×200. TUNEL-positive cells were quantified using image analysis software (image-Pro Plus 6.0).

### Immunofluorescence assay

The paraffin-embedded sections of the brain tissues (*N* = 5) were dewaxed to water, soaked in 3% hydrogen peroxide solution, washed with PBS, and the slices were incubated in 0.1 M sodium citrate solution. The slices were blocked in the diluted goat serum and incubated at 37°C for 30 min, followed by calcium ion probe (Beyotime Biotechnology, China) or ActA primary antibody (Proteintech Biotechnology, China) and incubated at 4°C overnight. The sections were washed with PBS and added with fluorescence-labeled secondary antibody (SA00013-1 (CoraLite488 – conjugated Affinipure Goat Anti-Mouse IgG), SA00013-2 (CoraLite488 – conjugated Affinipure Goat Anti-Rabbit IgG), SA00013-3 (CoraLite594 – conjugated Goat Anti-Mouse IgG), SA00013-4 (CoraLite594 – conjugated Goat Anti-Rabbit IgG), Proteintech, China). The sections were incubated at 37°C for 30 min, and then the sections were washed. The nuclei were stained with DAPI and washed at room temperature for 10 min. The anti-fluorescence quenching tablets were stored in PBS, and the pictures were taken and the treatment-associated changes were observed under fluorescence microscope (Olympus DX51 fluorescence microscope, Olympus, Tokyo, Japan) at ×200. The fluorescence intensity was analyzed using the ImageJ software.

### Golgi staining

The rats’ brain tissues (*N* = 5) were gently washed with saline and soaked completely in Golgi staining solution, then placed in a cool and ventilated place in darkness for 14 days. Then the hippocampus of brain tissues were soaked for three times with distilled water, and soaked in 80% glacial acetic acid overnight. Then the brain tissues were washed with distilled water and put into 30% sucrose. The tissue was cut into 100 μm by an oscillating slicer, affixed to a gelatin slide, and left to dry overnight in the dark. The tissue sections were treated with concentrated ammonia for 15 min, distilled water for 1min, acid film fixed solution for 15min, and distilled water for 3 min. After dried, the tissue sections were sealed with glycerin gelatin. Brightfield images for brain samples were taken by Olympus BX51 microscope (Olympus, Waltham, USA) at ×400. The microphotographs and spine density (the number of spines per 10 μm) were analyzed with Image Pro Plus 6.0 software (Media Cybernetics, Bethesda, MD, USA). Spines were counted by an experienced researcher blind to experimental design when manually regulated the focus to find all spines on a particular dendrite. Spine density was defined as the density of all spines counted per animal divided by the total length of the dendrite, which was demonstrated as the number of spines identified per μm dendrite [45].

### Western blotting assay

The brain tissue (*N* = 3) was homogenized, centrifuged at 12000 rpm at 4°C for 15 min. The supernatant was extracted and the protein concentration was examined by BCA method. The protein samples were loaded into SDS-PAGE electrophoresis, and transferred to PVDF membrane. The membrane was blocked in skim milk overnight. Then the primary antibodies including GAP-43(1:1000), SYN(1:1000), PSD-95(1:1000), MAP-2(1:1000), NMDAR(1:1000), NR2A(1:1000), NR2B(1:1000), BCL2(1:1000), BAX(1:1000), Wnt3a(1:1000), β-catenin(1:1000), VEGF(1:1000), CyclinD1(1:1000), GAPDH(1:1000) were incubated separately for 1 h. Following with that, the secondary antibody was added and incubated for 1 h. After ECL luminescence kit for visualization, the Image J software was performed for gray value analysis. primary antibodies purchased from Proteintech Biotechnology.

### Statistical analysis

SPSS19.0 statistical software (IBM) was performed for statistical analysis. The experimental data was expressed as mean ± standard deviation. The normality and homogeneity were checked using SPSS19.0 statistical software. One-way ANOVA with a turnkey post-test of variance was performed for analysis. Chi-square test was used for ratio comparison. *P* < 0.05 was considered statistically significant.

### Availability of data and materials

The analyzed datasets generated during the study are available from the corresponding author on reasonable request.
